# Injectable Hydrogel Delivery System with High Drug Loading for Prolonging Local Anesthesia

**DOI:** 10.1002/advs.202309482

**Published:** 2024-03-13

**Authors:** Yongchun Li, You Chen, Yifan Xue, Jinlong Jin, Yixin Xu, Weian Zeng, Jie Liu, Jingdun Xie

**Affiliations:** ^1^ Department of Anesthesiology Sun Yat‐Sen University Cancer Center State Key Laboratory of Oncology in Southern China Guangdong Provincial Clinical Research Center for Cancer Guangzhou Guangdong 510060 China; ^2^ School of Biomedical Engineering Shenzhen Campus of Sun Yat‐sen University Guangming District Shenzhen Guangdong 518107 China

**Keywords:** anesthesia, drug delivery, hydrogel, microsphere, peripheral nerve block

## Abstract

Peripheral nerve block is performed for precise pain control and lesser side effects after surgery by reducing opioid consumption. Injectable hydrogel delivery systems with high biosafety and moisture content have good clinical application prospects for local anesthetic delivery. However, how to achieve high drug loading and long‐term controlled release of water‐soluble narcotic drugs remains a big challenge. In this study, heterogeneous microspheres and an injectable gel‐matrix composite drug delivery system are designed in two steps. First, heterogeneous hydrogel microspheres loaded with ropivacaine (HMS‐ROP) are prepared using a microfluidic chip and in situ alkalization. An injectable self‐healing hydrogel matrix (Gel) is then prepared from modified carboxymethylcellulose (CMC‐ADH) and oxidized hyaluronic acid (OHA). A local anesthetic delivery system, Gel/HMS‐ROP/dexmedetomidine (DEX), with long‐term retention and drug release in vivo is prepared by combining HMS‐ROP and Gel/DEX. The drug loading of HMS‐ROP reached 41.1%, with a drug release time of over 160 h in vitro, and sensory and motor blockade times in vivo of 48 and 36 h, respectively. In summary, the sequential release and synergistic analgesic effects of the two anesthetics are realized using core‐shell microspheres, DEX, and an injectable gel, providing a promising strategy for long‐acting postoperative pain management.

## Graphical abstract

In this study, HMS‐ROP and an injectable gel‐matrix composite drug delivery system are designed in two steps. And then the composite drug delivery system with high drug loading releases DEX and ROP sequentially after being injected around the nerve to achieve long‐term local anesthesia effect.

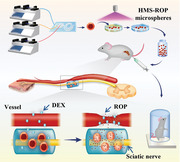

## 1 Introduction

Enhanced recovery after surgery (ERAS) is a multimodal multidisciplinary approach that can enhance recovery and reduce complications by using several care improvement processes.^[^ ^1^ ^]^ Among them, peripheral nerve block is critical for ERAS since it is a widely used clinical procedure that performs precise pain control and decreases nausea, vomiting, and the likelihood of respiratory depression due to the reduction of opioid consumption.^[^ ^2^ ^]^

Currently, local anesthesia is an effective strategy for perioperative pain management.^[^ ^3^, ^4^ ^]^ However, due to the high effective drug concentration required for anesthetic drugs, using traditional single‐injection anesthetic drugs exhibits significant limitations, including short effective analgesia and systemic toxicity.^[^ ^5^, ^6^ ^]^ To address these issues, clinical attempts have been made, such as by implanting catheters for continuous administration, which might inconvenient, expensive, technically demanding, and might increase the risks of infections and nerve injury. Therefore, recent investigations in local analgesia have focused on improving analgesic time and reducing the toxicity of anesthetics.

With the development of biomaterials, an increasing number of functional drug carriers have been developed for the controlled release and delivery of drugs. Liposomes, the earliest studied drug carriers, have the advantages of high biocompatibility and simple structure. Certain anesthetic liposomes, such as bupivacaine liposomes, have been approved for clinical use. However, the disadvantages of a complicated manufacturing process, obvious drug leakage, and short local analgesia times still exist.^[^ ^7^, ^8^, ^9^ ^]^ In addition, polymeric microspheres or nanoparticles are also widely studied as anesthetic delivery systems, including poly(lactic acid), poly(lactic‐co‐glycolic acid), and poly(ε‐caprolactone).^[^ ^10^, ^11^, ^12^, ^13^ ^]^ However, the local analgesia time of the above polymer‐basic anesthetic drug delivery systems rarely exceeds 24 h. Meanwhile, the clinical application of polymeric carriers remains limited, owing to the complicated preparation process, slow in vivo degradation rate, and easy induction of local inflammation. Most importantly, most local anesthetics used clinically are water‐soluble and are difficult to encapsulate in polymer particles.^[^ ^14^ ^]^ Moreover, although a large number of different drug carriers have been successfully developed, the drug loading of water‐soluble anesthetics is generally less than 20%.^[^ ^15^, ^16^ ^]^

Hydrogel‐based nanoparticles or microspheres, with network structures generated by crosslinking hydrophilic polymers, are widely used in injectable delivery applications. They can encapsulate a large amount of water and other substances and achieve water‐soluble drug release owing to their unique physicochemical properties and biocompatibility.^[^ ^17^, ^18^, ^19^, ^20^, ^21^ ^]^ The main obstacle involving loading water‐soluble drugs in hydrogel‐based microspheres is how to prevent drug leakage due to the good swelling ability of hydrogel, which might reduce drug loading and increase the risk of drug burst release in vivo. Hence, a potential strategy to improve drug stability and long‐term retention in vivo is to design a composite drug delivery system using drug‐loaded hydrogel microspheres and hydrogels.^[^ ^22^, ^23^ ^]^

ROP, an amide local anesthetic widely used in regional anesthesia, is considered the most promising local anesthetic because of its low central nervous and cardiac toxicities.^[^ ^24^, ^25^ ^]^ However, the analgesic effect of a single injection of ROP could be maintained for only 6–12 h, which is unable to meet the clinical needs of patients. Researchers found that the effectiveness of local anesthesia could be prolonged by combining anesthetics with other anti‐inflammatory or sedative drugs, such as bupivacaine and dexamethasone, ropivacaine and clonidine, and bupivacaine and dexmedetomidine; the anesthesia time of these drug combinations was enhanced compared to that of anesthetics alone.^[^ ^26^, ^27^, ^28^ ^]^ DEX is a highly selective *α*2‐adrenergic receptor agonist with various effects, such as sedation, analgesia, anti‐anxiety, inhibition of sympathetic nerve activity, mild respiratory depression, and improvement of hemodynamic stability.^[^ ^29^ ^]^ DEX can increase the onset time of anesthesia in peripheral nerve blocks and prolong the block time of the sensory and motor nerves.^[^ ^30^ ^]^ These results suggest that the combination of anesthetic and adjuvant drugs is an effective strategy for developing an injectable hydrogel‐based compound anesthetic delivery system with high drug loading, long‐term retention, and controlled drug release.

**Scheme 1:**
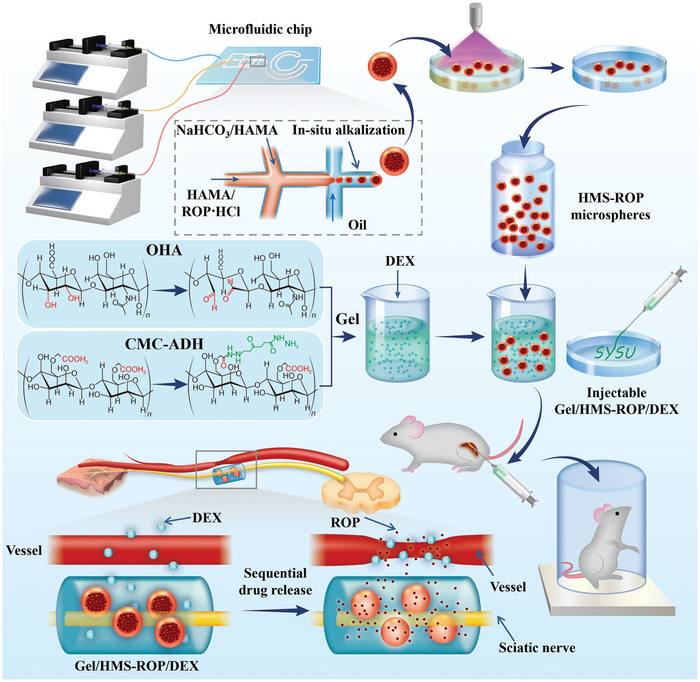
Schematic illustration of preparing a Gel/HMS‐ROP/DEX delivery system for prolonging the duration of regional nerve blockade.

In this study, we prepared uniformly sized drug‐loaded core‐shell hydrogel microspheres (HMS) based on high‐throughput microfluidic technology and in situ alkalization. Real‐time alkalization treatment and the core‐shell structure of HMS could realize stronger stability and higher drug loading of water‐soluble ROP. To improve the local long‐term retention of HMS at nerve sites in vivo, OHA and CMC‐ADH were used to prepare an injectable self‐healing hydrogel based on the Schiff base reaction. Finally, an injectable hydrogel‐based sustained‐release system Gel/HMS‐ROP/DEX was successfully constructed by co‐loading Gel/HMS‐ROP with the sedative drug DEX (**Scheme** **1**). The Gel/HMS‐ROP/DEX could be injected into nerve sites, forming a stable drug release reservoir, and achieving a synergistic anesthetic effect through the sequential release of the two drugs, eliciting 36 h of nerve block and 24 h of motor block. In addition, the Gel/HMS‐ROP/DEX exhibited good biodegradability and tissue compatibility. In conclusion, this system provides an effective approach for hydrogel‐based anesthetic drug delivery systems to achieve long‐term local anesthesia and analgesia.

## 2 Results and Discussion

### 2.1 Preparation and Characterization of the Injectable Hydrogel Matrix

**Figure 1:**
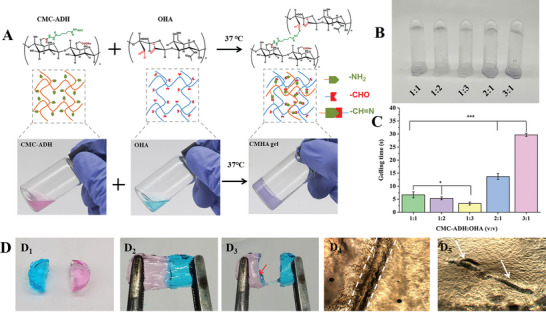
Preparation and gelation performance of the injectable self‐healing hydrogel. A) Schematic diagram and physical diagram of the crosslinking between CMC‐ADH and OHA. B) The gelation state and C) gelling time of the hydrogel were obtained using different ratios of CMC‐ADH and OHA. D) The self‐healing ability of the prepared hydrogel. (^*^ *p* < 0.05, ^***^ *p* < 0.001, n = 3).

In this study, we synthesized carboxymethy cellulose (CMC) and hyaluronic acid (HA) hydrogels with increasing ratios of amino and aldehyde groups, respectively. The ^1^H NMR spectra showed that ADH was successfully conjugated to CMC (CMC‐ADH) (Figure S1, Supporting Information). CMC‐ADH was mixed with OHA, resulting in a Schiff base reaction and the generation of dynamic chemical bonds,^[^ ^31^ ^]^ which endowed the hydrogel with good injectability and self‐healing capabilities (**Figure** **1**A). Stable hydrogels were formed by mixing CMC‐ADH and OHA in different proportions. The gelation time of the hydrogel was precisely controlled by adjusting the proportions of CMC‐ADH and OHA (Figure 1B). As shown in Figure 1C, the gelation time of the hydrogel was less than 30 s, which increased with an increase in CMC‐ADH and a decrease in OHA because the number of amino groups in CMC‐ADH was higher than the number of aldehyde groups in OHA.

CMC‐ADH and OHA were stained pink and blue, respectively, and the two different colored hydrogels were placed together to allow close contact. After 2 h at 25 °C, the two hydrogel blocks formed one block that could withstand tension perpendicular to the cutting surface without separation (Figure 1D_1‐3_). In addition, the self‐healing process of the cross‐section between the two hydrogel blocks was recorded using an optical microscope. Interestingly, the gap between the two hydrogel blocks was obvious at the initial stage but completely disappeared after 2 h (Figure 1D_4‐5_), and their self‐healing ability was higher than those reported in the literature.^[^ ^31^ ^]^ These results indicate that the hydrogel crosslinked with CMC‐ADH and OHA has an efficient self‐healing ability.

### 2.2 Preparation of HMS‐ROP Using a Microfluidic Chip

**Figure 2:**
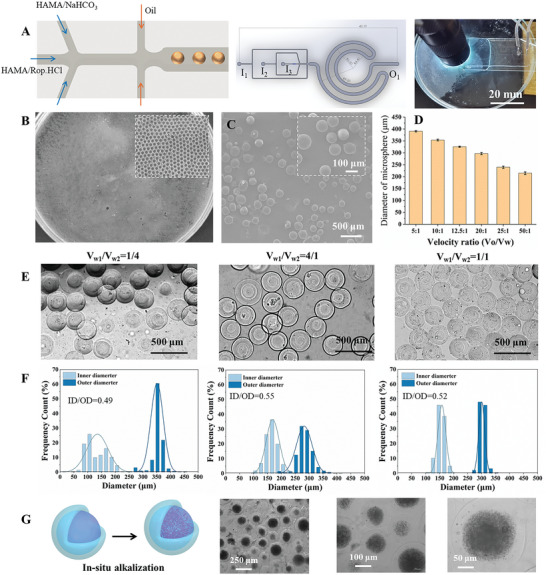
A) The schematic diagram for three‐channel chips and the preparation of the core‐shell microsphere (HMS‐ROP). B) Optical image and C) SEM image of HMS. D) The diameter of HMS is produced from different ratios of the velocities of the oil phase and water phase. E) Optical images and F) the inner/outer diameter of HMS prepared with different velocity ratios of the inner/outer water phase. G) Schematic diagram and optical images of the in situ alkalization of HMS‐ROP.

Drug‐loaded core‐shell hydrogel microspheres (HMS) with heterostructures were prepared using a three‐channel microfluidic chip. Photocrosslinked (methacryloyl hyaluronic acid) HAMA was successfully prepared (Figure S2, Supporting Information). As shown in **Figure** **2**A, there were three entrances (I_1_, I_2_, and I_3_) and one exit (O_1_) on the chip, where I_1_–I_3_ were the entrances of the oil phase, the outer water phase, and the inner water phase, respectively. First, the water phase consisting of HAMA was sheared by the oil phase to form droplets of controllable sizes. Second, the NaHCO_3_ in the outer water phase could penetrate the inner water phase, inducing the in situ alkalization of ROP·HCl, which recrystallizes the water‐soluble anesthetics into the lipophilic phase. Finally, HMS‐ROP was formed via UV irradiation photocrosslinking. The optical microscopy and scanning electron microscopy (SEM) images indicate that the microspheres prepared using the three‐channel chip had a uniform size and smooth surface (Figure 2B,C). The overall microsphere size was accurately controlled by adjusting the velocity ratios of the oil and water phases.^[^ ^32^ ^]^

As shown in Figure 2D, the size of microspheres could be adjusted from 200 to 375 µm and decreased by increasing the velocity of the oil phase, and the diameter of the obtained microspheres exhibited a normal distribution (Figure S3, Supporting Information). In addition, we investigated the impact of the velocities of the outer water phase (V_W1_) and inner water phase (V_W2_) on the size of the microspheres. The results in Figure 2E,F show that the outer/inner diameter ratio of HMS was 0.49, 0.55, and 0.52 when the ratio V_W1_:V_W2_ was 1:4, 4:1, and 1:1, respectively, indicating that adjusting the velocity ratio of outer/inner water phase had little effect on the size of microspheres, consistent with previous reports.^[^ ^33^ ^]^ Based on the above results, we used a V_W1_:V_W2_ ratio of 1:1 to prepare stable and uniform HMS‐ROP, which had average outer diameter and inner diameters of 328 ± 35 and 171 ± 46 µm, respectively (Figure 2F). The in situ alkalization of HMS‐ROP is shown in Figure 2G and Video S1 (Supporting Information). ROP·HCl was rapidly converted into ROP when droplets formed inside the chip (Figure S4, Supporting Information). After alkaline treatment, recrystallized ROP was obtained in the inner water phase and was surrounded by a shell with a thickness of ≈150 µm, which was beneficial to achieving the stable and long‐term drug release of HMS‐ROP.

### 2.3 Gelation and Injectability of Gel/HMS‐ROP

**Figure 3:**
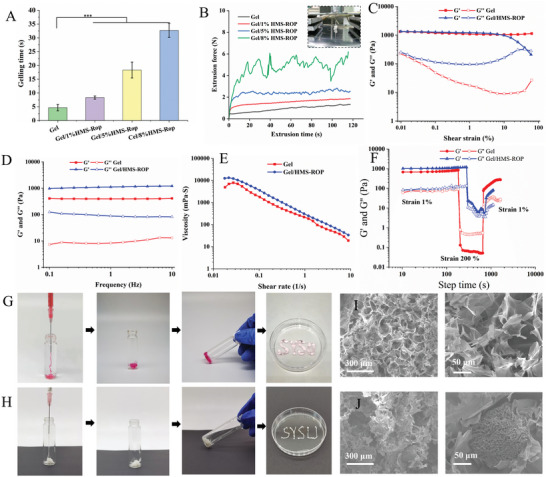
A) Gelation time of the different hydrogel formulations. B) Extrusion force of the Gel/HMS‐ROP composite with different formulations. C) The amplitude and D) frequency scanning results of the Gel and Gel/HMS‐ROP composite. E) The shear rate scanning results of the Gel and Gel/HMS‐ROP. F) The 3ITT test shows the self‐healing performance of Gel/HMS‐ROP composite. The injectability and SEM images of the Gel matrix (G, I) and Gel/HMS‐ROP composite (H, J). (^***^ *p* < 0.001, n = 4).

The loading proportion of HMS‐ROP was optimized based on the gelation and injectability of the Gel/HMS‐ROP composite. As shown in **Figure** **3**A, the gelation time was prolonged with increasing HMS‐ROP content. The gelling time was 8.3 ± 0.6, 18.3 ± 2.9, and 32.7 ± 2.5 s when the HMS‐ROP content was 1% (w/v), 5% (w/v), and 8% (w/v), respectively. Among them, the gelation time of Gel/8%HMS‐ROP was eight times longer than that of the pure hydrogel (4.7 ± 1.1 s). In the process of injection, the extrusion force of the HMS‐ROP with different concentrations was unstable, which shows that the HMS‐ROP microsphere are unevenly dispersed and prone to blockage during injection (Figure S5, Supporting Information). However, the Gel and HMS‐ROP composite exhibited a stable extrusion force during the injection process, indicated that the Gel material was benefit to improve the dispersibility and injectability of the HMS‐ROP microspheres. Although the extrusion force increased with increasing HMS‐ROP content, all values were less than 6 N (Figure 3B). As shown in Figure 3B, except for the Gel/8%HMS‐ROP group, the extrusion force of the other groups remained stable during the injection process, indicating that the addition of HMS‐ROP had a negative effect on the injectability of the hydrogel, but it could still be injected (Video S2, Supporting Information). The transparency of the hydrogels gradually decreased after the addition of HMS‐ROP (Figure S6A, Supporting Information). The mechanical strength of drug delivery system is closely related to the in vivo degradation, drug release, and neurocompatibility of the materials. With the increase of HMS‐ROP content, the compressive strength of Gel/HMS‐ROP decreased remarkably (Figure S6B, Supporting Information). The compressive strength of Gel and Gel/8%HMS‐ROP were 80 ± 10.8 and 26.6 ± 3.3 kPa, respectively. Therefore, the compressive strength could be precisely controlled by adjusting the ratio of the Gel and HMS‐ROP materials. In fact, the drug delivery system with low mechanical strength not only have good compatibility with tissues, but also have better drug controlled release ability. Therefore, the Gel/8%HMS‐ROP composite matched the requirements of gelation, injectability, and drug‐loading capacity and was selected for subsequent experiments.

### 2.4 Rheological Evaluation of the Gel/HMS‐ROP Composite

The rheological properties of the gels were also evaluated. The results of amplitude scanning are shown in Figure 3C. There was no significant difference in the storage modulus (G') of the Gel and Gel/HMS‐ROP composite. The G' of Gel did not change significantly with the increase of oscillation strain, but the G' of Gel/HMS‐ROP decreases rapidly when the oscillation model exceeds 10%. It was found that the G' and loss moduli (G″) of the Gel/HMS‐ROP composite were the same when the oscillation strain was 40%. This indicates that the Gel/HMS‐ROP composite transformed from the gel state to the flow state while the gel structure remained stable. The above results revealed that gels with higher structural stabilities could bear greater shear stress and that the structure of Gel/HMS‐ROP changed when the degree of shear was large, which was beneficial for improving the injectability of the material.^[^ ^34^ ^]^ The frequency scanning results (Figure 3D) showed that the storage modulus of the Gel and Gel/HMS‐ROP composite groups were higher than its loss modulus and changed slightly with increasing frequency, indicating that the material had a stable crosslinking network.^[^ ^35^ ^]^ Moreover, shear rate scanning (Figure 3E) showed that the viscosity of the Gel/HMS‐ROP composite was slightly higher than that of the Gel alone. The viscosity of the Gel and Gel/HMS‐ROP composite groups decreased with an increase in shear rate, demonstrating that the material possessed preferable injectability owing to its better shear thinning behavior.^[^ ^36^ ^]^

Figure 3F shows the results of 3ITT test for Gel and Gel/HMS‐ROP. The G' value was higher than the G″ value when the 1% strain in the whole first stage. However, a sharp decrease in the G' and G″ value was observed in first stage when a high shear strain (200% strain) was conducted to the materials. At the last stage, the G' and G″ value could be recovered when the same shear train as first stage was applied. In the third stage, the G' value of Gel (320 ± 1.6 Pa) and Gel/HMS‐ROP (74.7 ± 4.8) recovered to 42% and 12% of their initial values respectively within 15 min, which was attribute to the reversible weak interaction of hydrogen bonds in hydrogels.^[^ ^37^ ^]^ These results suggested that the Gel and Gel/MS‐ROP samples have self‐healing ability, which is beneficial for the materials to form stable hydrogels again after injection into the body and better serve as drug reservoirs.

### 2.5 Morphology of the Gel/HMS‐ROP Composite

Figure 3G,H shows that the Gel and Gel/HMS‐ROP composite had good injectability, as they could be injected in a continuous filamentous structure. The post‐injection SEM images showed a porous network structure in the Gel group, owing to the pores generated by the freeze‐drying of water (Figure 3I). The pores of the Gel/HMS‐ROP group with HMS‐ROP were uniformly distributed inside the gel matrix material (Figure 3J) and were smaller than those of the Gel group, indicating that the diameters of the pores of the gel matrix material decreased with the addition of HMS‐ROP. This would be beneficial in reducing the rapid diffusion of ROP in the hydrogel, prolonging the drug release time.

### 2.6 Drug Loading and Drug Release

**Figure 4:**
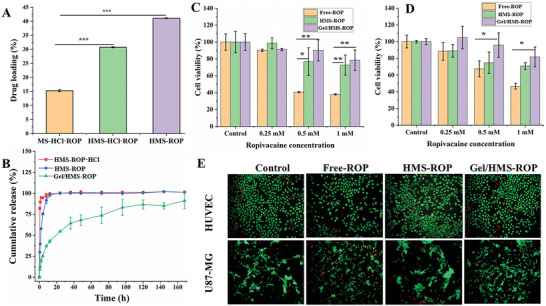
A) The drug‐loading capacities of the different formulations. B) The time‐dependent drug release of the different formulations. C) The viability of human umbilical vein endothelial cells (HUVECs) and D) U87‐MG cells after incubation with the different formulations. E) Results of the Live/Dead staining of HUVECs and U87‐MG cells after incubation with the different formulations. (^*^ *p* < 0.05, ^**^ *p* < 0.01, ^***^ *p* < 0.001, n = 4).

In order to meet the requirement of the pain control, local anesthetics always require a high anesthetic concentration and a long sustained release time. Hence, a controlled, sustainable drug delivery system is necessary to achieve a loading capacity with a high initial drug dose. As shown in **Figure** **4**A, the drug‐loading capacities of MS‐ROP·HCl, HMS‐ROP·HCl, and HMS‐ROP were 15.3% ± 0.4, 30.7% ± 0.2, and 41.1% ± 0.1, respectively. Specifically, the drug‐loading capacity of HMS‐ROP·HCl was two times higher than that of MS‐ROP·HCl, indicating that the loading of water‐soluble drugs could be significantly improved by a heterogeneous core‐shell microsphere design. This is because the shell layer can reduce the diffusion rate of the drug. Moreover, after in situ alkalization treatment, the drug loading of HMS‐ROP was further improved by 2.7 times and 1.3 times higher than that of MS‐ROP·HCl and HMS‐ROP·HCl, respectively; this was significantly higher than most of the hydrogel microspheres (less than 10%) reported in the literature.^[^ ^38^, ^39^ ^]^ Notably, HMS‐ROP is the microsphere carrier with the highest drug‐loading capacity for local anesthesia.^[^ ^16^, ^28^, ^40^ ^]^ In summary, using microfluidic technology and in situ alkalization, the HMS‐ROP drug‐loading system could achieve high drug‐loading capacity. In addition, preparation of core‐shell microspheres based on microfluidic chip technique is expected to be a general strategy to improve the loading capacity of water‐soluble drugs.

Limited burst release and the prolonged, sustainable release of drugs are important parameters for evaluating drug carriers. Figure 4B shows that the accumulated drug release of HMS‐ROP·HCl, HMS·ROP, and Gel/HMS‐ROP in 0.5 h were 82.4%, 29.69%, and 9.48%, respectively; HMS‐ROP·HCl exhibited a significant burst release of the drug. In comparison, Gel/HMS‐ROP exhibited a sustained release of the drug for over 168 h. These results suggest that the water‐soluble drugs loaded in the hydrogels were released quickly, owing to the rapid swelling performance of the hydrogel. However, the burst release and sustained release duration both improved significantly after in situ alkalinization treatment. Moreover, the drug release performance of the microspheres was further improved by compounding the hydrogel matrix with drug‐loaded microspheres, indicating that this system is a potential strategy for developing an efficient controlled release system for drugs.

### 2.7 In Vitro Cell Compatibility

Systemically administered DEX has been proved to have anesthetic effect without neurotoxicity.^[^ ^28^, ^41^ ^]^ Therefore, the cytotoxicity of ROP has been focused on in this study. Owing to the cytotoxicity of ROP against nerve cells, developing a carrier system needs to control drug release and reduce side effects. We then evaluated the cytotoxicity of the composite gel sustained‐release system in HUVECs and U87‐MG cells. As shown in Figure 4C and 4D, free ROP exhibited a concentration‐dependent cytotoxicity against HUVECs and U87‐MG cells, with cell viability of 38.1% and 46.7%, respectively, after treatment with 1 mm ROP. Meanwhile, the cell viability in the HMS‐ROP and Gel/HMS‐ROP groups at the same drug concentrations exceeded 70% and 80%, respectively. These results suggest that the HMS and the gel‐matrix composite reduced the cytotoxicity of ROP. The Live/Dead staining results of HUVECs and U87‐MG cells incubated with different formulations (0.5 mm) are shown in Figure 4E, which were consistent with the cytotoxicity assay results. Furthermore, the cell morphology in the Gel/HMS‐ROP group was similar to that in the control group. These results suggest that Gel/HMS‐ROP can significantly reduce the side effects compared to the free drugs.

### 2.8 Pharmacodynamic Evaluation In Vivo

**Figure 5:**
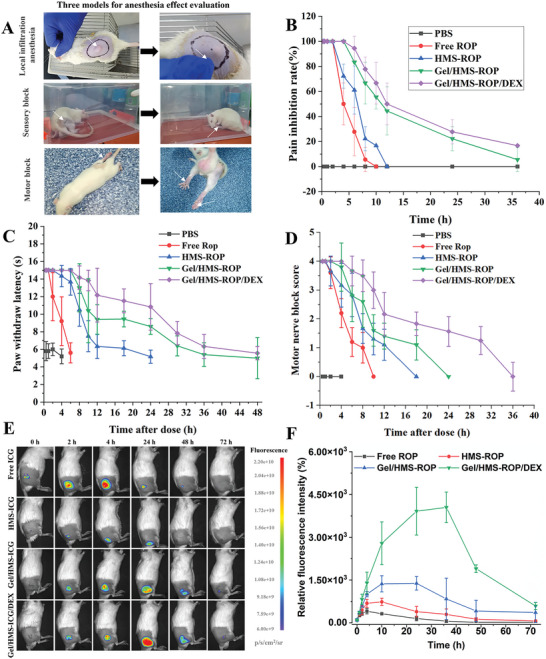
A) The local infiltration anesthesia, sensory block, and motor block in vivo models were used to verify the drug efficacy. B) The pain inhibition rate in SD rats after different treatments at various times. C) Sensory blockade was evaluated using the hot plate test. D) The motor block effect score at different times after different treatments. E) In vivo fluorescence imaging of the different formulations in SD rats at different time intervals after injection. F) Semi‐quantitative fluorescence intensity analysis of the injection site at different times.

In this study, the local infiltration anesthesia, sensory block, and motor block in vivo models were used to verify the drug efficacy (**Figure** **5**A). The body weight of the Sprague‐Dawley (SD) rats used in the in vivo model was ≈250–275 g, with small differences between groups (Figure S7, Supporting Information). First, the local infiltration pharmacodynamics of the different formulations were evaluated using a local infiltration anesthesia model. As shown in Figure 5B, the pain inhibition effect in all groups decreased over time and the time when the pain inhibition rate exceeds 50% was defined as an effective pain inhibition. The effective pain inhibition rates of the free ROP, HMS‐ROP, Gel/HMS‐ROP, and Gel/HMS‐ROP/DEX were 4, 6, 10, and 12 h, respectively. The local analgesic time of the Gel/HMS‐ROP/DEX group and HMS‐ROP group was 3 and 1.5 times higher than that of the free ROP group, respectively, indicating that the microspheres and hydrogel matrix could significantly improve the analgesic duration of ROP.

Furthermore, we evaluated the in vivo the ansethetic effect of sciatic nerve blockade after treatment with the different formulations, which were detected using a hot plate test. After injection with the different formulations, the toes of the rats showed a contractive state without feeling pain on the hot plate during the sensory blockade, and obvious licking or jumping behavior occurred when the anesthetic effect was insufficient. As shown in Figure 5C, the effective nerve block time was defined as exceeding 10 s, the effective nerve block time of the Gel/HMS‐ROP group (24 h) was significantly higher than that of the HMS‐ROP group (10 h), and the free ROP group (4 h), which was six times longer than that of the free ROP group. Interestingly, the sensory block time of Gel/HMS‐ROP/DEX group was the same as that of Gel/HMS‐ROP, but its pain response time was obviously lower than that of Gel/HMS‐ROP. In addition, the effects of the nerve block in the Gel/HMS‐ROP/DEX and Gel/HMS‐ROP groups decreased slowly. In contrast, the nerve block effect score of the HMS‐ROP and free ROP groups decreased rapidly with time. This indicates that the hydrogel and microspheres could significantly improve the nerve block effect of ROP in vivo and that the addition of DEX could prolong the anesthetic effect of ROP in the sensory nerve block, owing to the vasoconstrictive and sedative effects of DEX to reduce the drug metabolic rate.^[^ ^28^ ^]^

The motor‐blocking effects of different formulations were also evaluated. Two hours after injecting the different formulations, rats with hind limb paralysis and toe contraction were unable to perform normal flexion, extension, and movement and lacked normal motor function (Video S3, Supporting Information). The maximum motor block times of Gel/HMS‐ROP/DEX, Gel/HMS‐ROP, HMS‐ROP, and free ROP were 36, 24, 18, and 10 h, respectively, indicating that the microspheres and hydrogel matrix improved the motor block effect of ROP and adding DEX further enhanced this effect (Figure 5D). Interestingly, the nerve block time of Gel/HMS‐ROP/DEX (48 h) was higher than its motor block time (36 h), confirming that the nerve and motor block effects of ROP were separate, which allowed the rat to move effectively without feeling pain. The difference between the time of sensory block and motor block was mainly attributed to the mechanism of ROP, the drug has the effect of separating motor block from nerve block, which is a favorable result for local anesthesia. Effective pain relief and early mobilization are essential for fast‐track rehabilitation and be in line with principles of ERAS. It was demonstrated that sensory selectivity of Gel/HMS‐ROP/DEX posted a prospect for walking regional analgesia.

### 2.9 In Vivo Drug Release

To further investigate the drug release performance in vivo, the Gel/HMS was marked with a water‐soluble near‐infrared fluorescent dye (indocyanine green, ICG). Owing to the aggregation‐induced quenching effect of ICG, the fluorescence signal of ICG increases when the drug is released from the carrier; therefore, the release of ICG could be dynamically monitored from the change in the ICG fluorescence signal. In addition, the concentration of water‐soluble DEX is as low as nanogram, and the drug is released quickly in vivo, so it is difficult to detect the accurate sustained drug release. However, it has been proved that DEX can reduce the metabolism of narcotic drugs and improve the release time of narcotic drugs in vivo.^[^ ^28^ ^]^ As shown in Figure 5E, the fluorescence signal in the free ICG group increased and then decreased rapidly, whereas HMS‐ICG, Gel/HMS‐ICG, and Gel/HMS‐ICG/DEX exhibited delayed release. ICG fluorescence signals in the Gel/HMS‐ICG and Gel/HMS‐ICG/DEX groups could still be observed at 72 h. The results of the semi‐quantitative analysis of fluorescence intensity are shown in Figure 5F. The relative fluorescence intensity of the Gel/HMS‐ICG/DEX group was significantly higher than that of the other groups, indicating that adding DEX reduced drug absorption and prolonged the drug release time through vasoconstriction.^[^ ^28^, ^41^ ^]^

### 2.10 Biosafety Evaluation of the Composite

**Figure 6:**
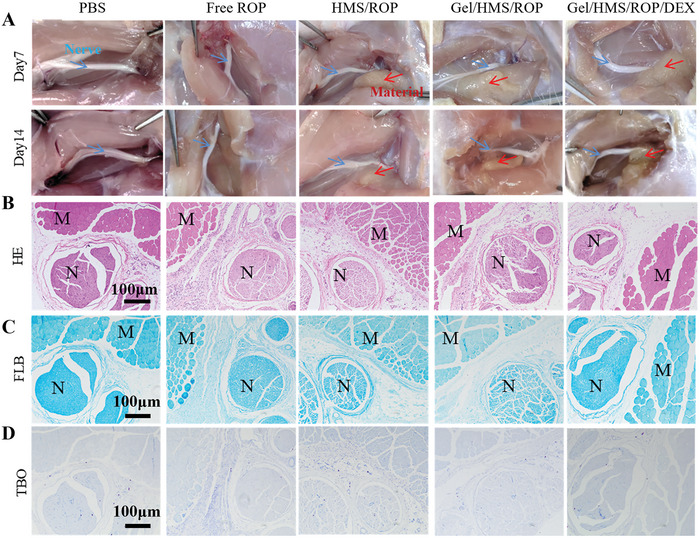
A) Anatomical images of the rats after treatment with the different formulations after 7 and 14 days. B) H&E, C) FLB, and D) TBO staining results of the nerve site after the different treatments. Red arrows indicate the materials and blue arrows indicate the nerves. N and M represent sciatic nerve, muscle, respectively.

**Figure 7:**
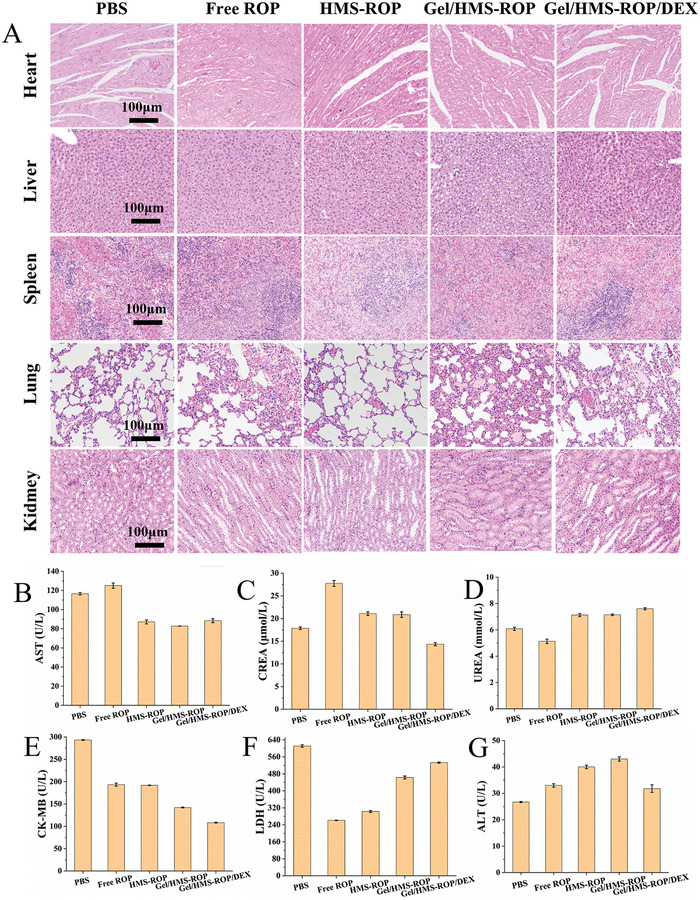
A) H&E staining of the main organs (heart, liver, spleen, lung, and kidney) after the different treatments. B–G) Levels of serum biochemical indicators (AST, CREA, UREA, CK‐MB, LDH, and ALT) after the different treatments.

At 7 and 14 days after the injection of the different formulations, the in vivo neural integrity and degree of material degradation were observed by dissecting the injection site. As shown in **Figure** **6**A, the nerves in all groups maintained their integrity, and the materials exhibited a gradual degradation trend. Hematoxylin and eosin (H&E), Fast Blue (FLB), and Toluidine Blue O (TBO) staining showed that the muscles and nerves remained intact after the injection of the treatments and that Gel/HMS‐ROP/DEX did not induce local tissue toxicity (Figure 6B–D). Furthermore, as shown in **Figure** **7**A, almost no inflammation or other pathological changes in the heart, liver, spleen, lungs, and kidneys were observed in any of the groups, demonstrating the satisfactory biosafety and biocompatibility of the composite. Moreover, the levels of biochemical indices such as AST, CREA, UREA, CK‐MB, LDH, and ALT were maintained in the normal range compared with the control group, indicating the safety of Gel/HMS‐ICG/DEX (Figure 7B–G).

## 3 Conclusion

In this study, a Gel/HMS‐ROP/DEX composite hydrogel delivery system consisting of heterogeneous core‐shell microspheres loaded with ROP, an injectable self‐healing gel matrix, and the anesthesia adjuvant DEX was prepared. First, the drug was brought to the periphery of the nerve, and a drug depot was formed. A long‐term anesthetic effect was realized through the sequential and controlled release of DEX and ROP through the microspheres and gel matrix. Compared to the free ROP group, the drug release time in vitro and nerve block time in vivo in the Gel/HMS‐ROP/DEX group were significantly improved by the synergistic effect of the drugs. In addition, the drug delivery carriers were biodegradable in vivo and did not damage the peripheral nerve tissue. These results collectively suggest that the hydrogel drug delivery system fabricated in this study is a promising anesthetic drug carrier for achieving long‐term local analgesia.

## 4 Experimental Section

### Materials

Polydimethylsiloxane (PDMS) was obtained from Dow Corning. Dexmedetomidine hydrochloride (DEX·HCl), mineral oil, ropivacaine hydrochloride (ROP·HCl), periodate, EDC, NHS, 2‐hydroxy‐4′‐(2‐hydroxyethoxy)‐2‐methylpropiophenone (2959), adipic dihydrazide (ADH), carboxymethyl cellulose (CMC), methacrylic anhydride (MA), and hyaluronic acid (HA) were obtained from Shanghai Macklin Biochemical Technology Co., Ltd. Span 80 and lithium phenyl‐2,4,6‐trimethylbenzoylphosphinate (LAP) were purchased from Sigma–Aldrich (St. Louis, MO, USA).

### Preparation of the Microfluidic Chip

The three‐channel microfluidic chip used in this study was designed and manufactured according to a previous report.^[^ ^42^ ^]^ In brief, the 3D modeling software SolidWorks was used to design the chip structure, and a male mold of the chip was prepared on a copper plate using a precision CNC machine (CNC, DS3030; Dingsheng Equipment Company, China). Subsequently, the male mold was washed three times with anhydrous ethanol. After drying, the surface of the male mold was covered with the PDMS, subjected to vacuum defoaming for 10 min, and cured overnight in an 80 °C oven. The PDMS chip was punched after stripping and trimming the template. Finally, the PDMS chip was bonded to a glass sheet via plasma treatment and stored after high‐pressure steam sterilization.

### Modification of Natural Polymer Materials

HAMA was prepared based on a previous study.^[^ ^43^ ^]^ In brief, 2 g HA (300 kDa) was added to 100 mL deionized water, and 1 mL MA was slowly added dropwise into the 2% HA solution. The reaction mixture was stirred in an ice bath for 24 h and maintained at pH 5.0 by adding a 5 m(mm) NaOH solution. The product was dialyzed at 4 °C for 3 days, and HAMA was obtained after freeze‐drying.

### CMC‐ADH

CMC‐ADH was also prepared based on a previous study.^[^ ^44^ ^]^ CMC (4.6 mmol) was dissolved into PBS (pH 4–5) at 50 °C. After cooling to 25 °C, EDC and NHS were added, the carboxyl groups were activated for 30 min, and 5% ADH was added to the solution, stirring for 24 h. The product was dialyzed for 3 days, and CMC‐ADH was obtained after freeze‐drying.

### OHA^[^ ^19^ ^]^

OHA was prepared based on a previous study.^[^ ^44^ ^]^ A 2% periodic acid solution was added to a 1% HA solution (in PBS, pH 5.0) and stirred at room temperature for 5 h in the dark. Ethylene glycol was then added to quench the reaction. The crude product was dialyzed for 3 days, and OHA was obtained after freeze‐drying. The structures of all the polymers were characterized using ^1^H NMR (Bruker Avance 400 MHz, Germany).

### Preparation of Drug‐Loaded Hydrogel Microspheres

Hydrogel microspheres loaded with ROP with a heterogeneous structure (HMS‐ROP) were prepared using a microfluidic chip. HAMA, ROP·HCl, and 2959 were dissolved in deionized water as the internal aqueous phase (core) at concentrations of 1% (w/v), 5% (w/v), and 0.5% (w/v), respectively. Similarly, 1% (w/v) of HAMA and 0.25% (w/v) of LAP were dissolved into a 0.1 m NaHCO_3_ solution to create the external aqueous phase (shell). The oil phase consisted of mineral oil containing 1% Span 80 surfactant. HMS‐ROP was prepared using a three‐channel microfluidic chip. The velocities of the external aqueous phase (V_W1_), internal aqueous phase (V_W2_), and the oil phase were 8.0, 8.0, and 50.0 µL min^−1^, respectively. The microspheres were crosslinked using a 365‐nm laser after outflowing the chip, and a 100‐µm filter was used to collect the microspheres from the oil phase. The microspheres were then rinsed once with petroleum ether and then thrice with PBS. The microspheres were collected after freeze‐drying and stored after sterilization. Finally, the size and morphology of the microspheres were characterized using optical microscopy (MXH‐100, Motic, China) and scanning electron microscopy (SEM; Quanta 400F, FEI, USA).

### Preparation and Characterization of Gel/HMS‐ROP/DEX Composites

A 3% (w/v) CMC‐ADH solution and 3% (w/v) OHA solution were mixed at a volume ratio of 1:1 to prepare a hydrogel precursor solution. Next, 8% HMS‐ROP and 20 µg mL^−1^ DEX were added rapidly to the precursor solution, and then the Gel/HMS‐ROP/DEX was obtained via gelation at 37 °C for 5 min after stirring uniformly by vortex oscillator. The rheological properties of the Gel/HMS‐ROP/DEX composite were characterized using a Haake rotational rheometer (HAAKE MARS III; Thermo Scientific, USA). Briefly, each sample was added to a rheometer plate (diameter, 25 mm; plate spacing, 0.5 mm). The equipment parameters were set as follows: 1) Amplitude scanning: test temperature 25 °C, oscillation frequency 1 Hz, oscillation strain range 0.01–100%; 2) Frequency sweep: oscillation frequency range 0.1–10 Hz, shear strain 0.1%; and 3) Shear rate scanning: test temperature 25 °C, oscillation frequency 1 Hz, shear rate range 0.01–10 s^−1^. The self‐healing performance of Gel/HMS‐ROP was evaluated by the three intervals thixotropy test (3ITT), which gave information about the degree of recovery after deformation.^[^ ^45^ ^]^ Briefly, the first interval was performed under a low shear strain of (1%), and then a high shear strain (200%) was conducted. At the third interval, the parameters was set to the same as those of the first interval.

Injectability can be determined by detecting the injection force during the extrusion process.^[^ ^46^ ^]^ Briefly, the sample was loaded into a print cartridge (27 G nozzle, 260 µm), then the influence of extrusion rate on the extrusion force of different proportions of composite biological ink was tested on a universal mechanical testing machine (5567, Instron, USA). The Gel/HMS‐ROP/DEX composite was subjected to gradient dehydration, freeze‐drying, and quenching in liquid nitrogen to obtain cross‐sectional structures. The morphology of the composite was observed via field‐emission SEM after spraying with gold.

### Drug Loading and Drug Release In Vitro

The drug‐loading capacity of the microspheres was detected as previously described.^[^ ^16^ ^]^ HMS‐ROP (20 mg) was soaked in methanol and fully dissolved. After centrifugation at 2000 rpm for 10 min, the supernatant was obtained, and the drug content was determined using high‐performance liquid chromatography (HPLC; LC‐2030, Shimazu, Japan). The HPLC system was equipped with a Venusil MP C18 column (4.6 mm × 250 mm, 5 µm) with acetonitrile: water (80:20, v/v) as the mobile phase. The column temperature and detection wavelength were set to 35 °C and 240 nm, respectively. In vitro drug release was detected as follows. Briefly, free ROP, HMS‐ROP, and Gel/HMS‐ROP (equivalent to 10 mg of ROP) were added to dialysis bags (14 kDa). The dialysis bags were then put into 20 mL of 0.02% Tween‐80 in PBS and incubated at 37 °C under 120 r min^−1^ oscillation. Finally, ROP release was detected using HPLC.

### Cytotoxicity Assays

Human umbilical vein endothelial cells (HUVECs) and U87‐MG cells were individually seeded into 48‐well plates at a density of 5 × 10^3^ cells per well. After incubation for 24 h, a fresh medium (DMEM) containing materials (Free‐ROP, HMS‐ROP, and Gel/HMS‐ROP) at different concentrations was added to the wells, and the group without materials was considered the control group. After incubation for 48 h, the spent medium was removed, and then a fresh medium containing 10% CCK8 assay solution was added to each well. Cell viability was determined using a microplate reader (BioTek Synergy 4; BioTek, USA). Finally, the cells were also treated with a Live/Dead Cell Staining Kit and observed using fluorescence microscopy.

### Local Infiltration Anesthesia Model In Vivo

Adult male SD rats were purchased from the Institute of Experimental Animals at the Guangdong Medicine Experimental Animal Center. All animal‐related processes were approved by the Sun Yat‐sen University Cancer Center Animal Care and Use Committee (No.L025501202303009). SD rats were randomly divided into five groups, with three rats per group, and hair was removed from the backs of the rats along the midline (6 cm × 8 cm). The rats were divided into five groups: PBS group (control group), 0.6% (w/v) ROP·HCl (positive control group), and 8% (w/v) HMS‐ROP, Gel/HMS‐ROP (containing 8% [w/v] HMS‐ROP), and Gel/HMS‐ROP/DEX (containing 8% [w/v] HMS‐ROP and 20 µg mL^−1^ DEX). The rats were subcutaneously injected with 500 µL of each treatment in a marked area, and the pharmacodynamic detection area was drawn around the injection area with a radius of 2 cm. The skin inside the detection area was punctured with a sterile injection needle at different time points, six times at each time point, for a 3–5 s time travel between two acupuncture needles, and the skin contraction in the area was negatively correlated with the effect of the local anesthesia. The number of times the rats exhibited no response was recorded, and the percentage of pain inhibition was calculated using the following equation: (number of times of no contraction reaction/6) × 100%.^[^ ^47^ ^]^

### Sciatic Nerve Blockade Model In Vivo

SD rats were randomly divided into five groups, with five rats per group. All rats were anesthetized with 2% isoflurane in oxygen and received left peri‐sciatic nerve injection. A 23G needle with a syringe was inserted into the posteromedial of the greater trochanter and advanced in an anteromedial direction, from posteromedial and anteromedial parallel to the spine. Once the needle made bone contact, 500 µL of each treatment was injected around the nerve. The sensory and motor functions of the rats were then evaluated using a hot plate and scale scoring at different time points. The thermal noception of the sciatic nerve block was evaluated using a self‐made 56 °C hot plate. The time from the outset of the thermal stimulus resulting in licking, withdrawing paw, or bouncing was recorded as a rat's paw withdrawal latency(PWL).^[^ ^48^ ^]^ First, the basic thermal latency of the rats was tested, and the value was set to 5 s. Second, rats with higher or lower base values were excluded. To protect the animals, the maximum PWL was set at 16 s. Motor function was evaluated using a digital scoring system based on animal behavior at different time points.^[^ ^28^ ^]^ The scoring system was as follows: normal movement (0 points); weight‐bearing activity, inability of toes to fully expand when raised by the tail (1 point); inability to fully load movement, inability of toes to expand (2 points); leg weakness, complete inability to bear weight, complete inability to open toes (3 points); and complete inability to bear weight and bend toes, and gait dragging (4 points).

### In Vivo Drug Release

ROP was replaced with ICG to evaluate the in vivo drug release from Gel/HMS‐ROP/DEX. Briefly, the rats were divided into four groups, with three rats in each group, and 0.5 mL free ICG, HMS‐ICG, Gel/HMS‐ICG, and Gel/HMS‐ICG/DEX were injected into the nervous system of each group. The fluorescence signal was observed ancquantified by using an Animal Living lmaging System (AniView Phoenix, China) at different timepoints. The amount of drug released in different groups was substituted by fluorescence intensity and high fluorescence intensity meaning more drugs were released.

### Biosafety Evaluation

The perineural tissue of the rats was fixed with a 4% (w/v) paraformaldehyde solution, embedded in paraffin, sectioned, and stained with H&E, TBO, and FLB. The main organs, including the heart, liver, spleen, lungs, and kidneys, were fixed with a paraformaldehyde solution and stained with H&E. For serum biochemistry analysis, the blood from each group of rats was centrifuged (1000 ×*g*, 4 °C, 10 min) to isolate the serum, and the levels of various biochemical indicators (LDH, AST, ALT, UREA, CK‐MB, and CREA) were detected using a blood biochemical analyzer (Chemray 800, Rayto, China).

### Statistical Analysis

All the data were presented as the mean ± standard deviation (SD) and use at least three samples for each data. Data were analyzed using one‐way analysis of variance (ANOVA) followed by Tukey's test using SPSS 19.0 software. Differences were considered statistically significant at *p* < 0.05.

## Conflict of Interest

The authors declare no conflict of interest

## Supporting information

Supporting Information

Supplemental Video 1

Supplemental Video 2

Supplemental Video 3

## Data Availability

The data that support the findings of this study are available in the supplementary material of this article.
