# Supplementary material for: Injectable Hydrogel Delivery System with High Drug Loading for Prolonging Local Anesthesia
Source: Adv Sci (Weinh). 2024 Mar 13;11(24):2309482. doi: 10.1002/advs.202309482 (PMC11200007; doi:10.1002/advs.202309482)
Supplement: Supplementary file 1 — Supporting Information [file ADVS-11-2309482-s001.pdf]

ADVANCED SCIENCE
Open Access

Supporting Information

for *Adv. Sci.*, DOI 10.1002/advs.202309482

Injectable Hydrogel Delivery System with High Drug Loading for Prolonging Local Anesthesia

Yongchun Li, You Chen, Yifan Xue, Jinlong Jin, Yixin Xu, Weian Zeng, Jie Liu and Jingdun Xie**

Support information

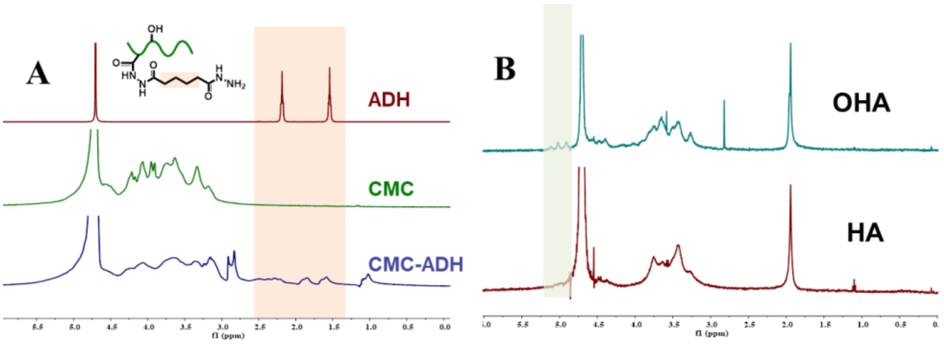

Figure.S1 [1] H-NMR spectra of (A) carboxymethyl cellulose (CMC), (B) hyaluronic acid (HA) and its modified products.

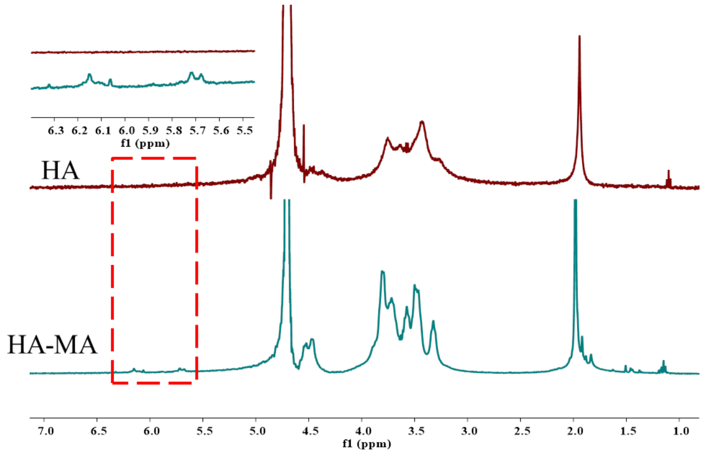

Figure.S2 [1] H-NMR spectra of (A) HA and (B) methacrylic anhydride modified HA (HAMA).

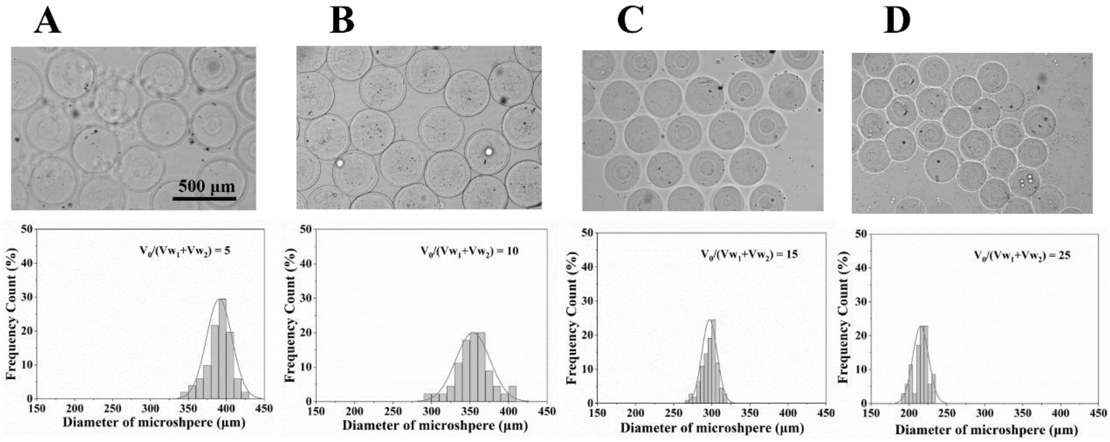

Figure.S3 Size and distribution of hydrogel microspheres prepared by different oil-water ratios based on microfluidic chip.

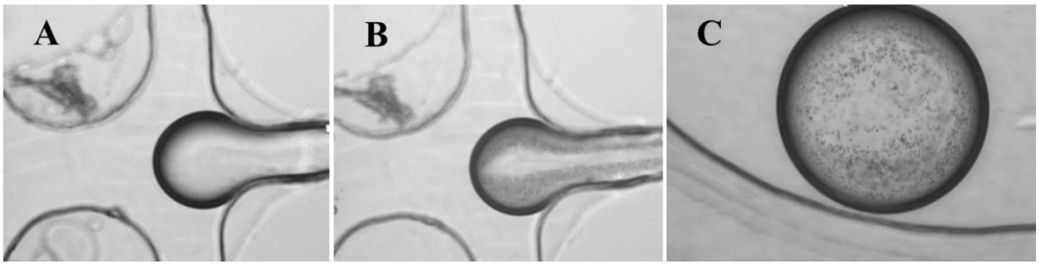

Figure.S4 Untreated and in-situ alkalization process in preparation of drug-loaded gel microspheres.

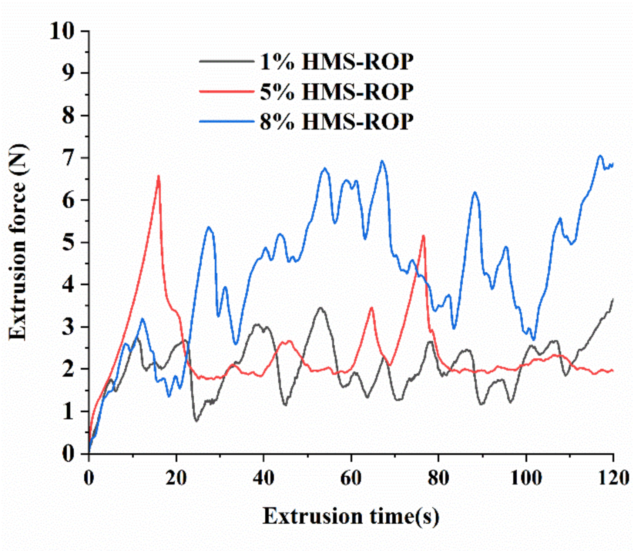

Figure.S5 The extrusion force of Gel and HMS-ROP groups showed a unstable extrusion force during the injection process.

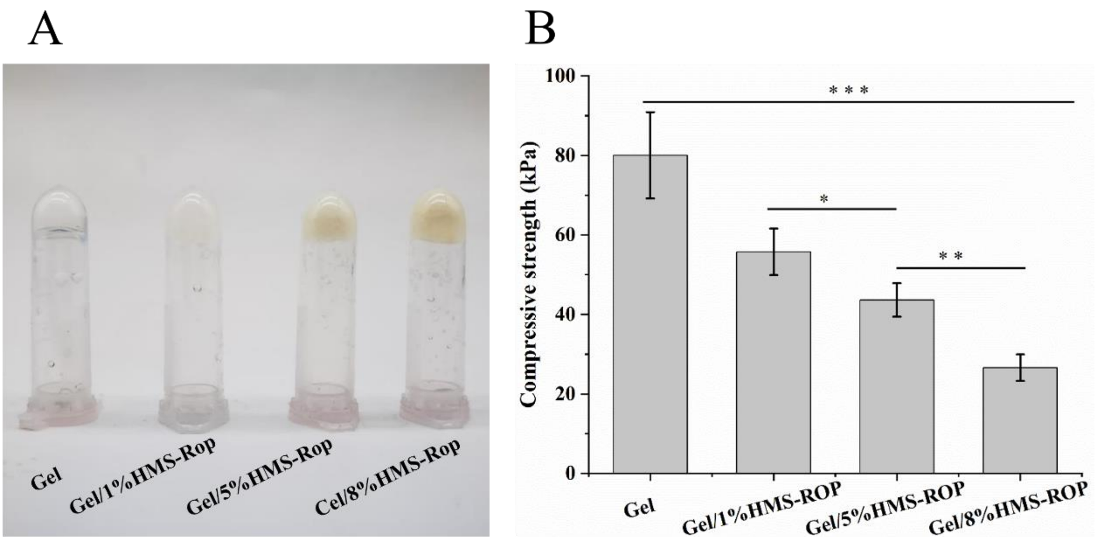

Figure.S6 (A) The optical images of the Gel and Gel/HMS-ROP groups and (B) their compressive strength was characterized by the universal mechanical experimental machine. (*P<0.05, ** P <0.01, *** P <0.001, n=4)

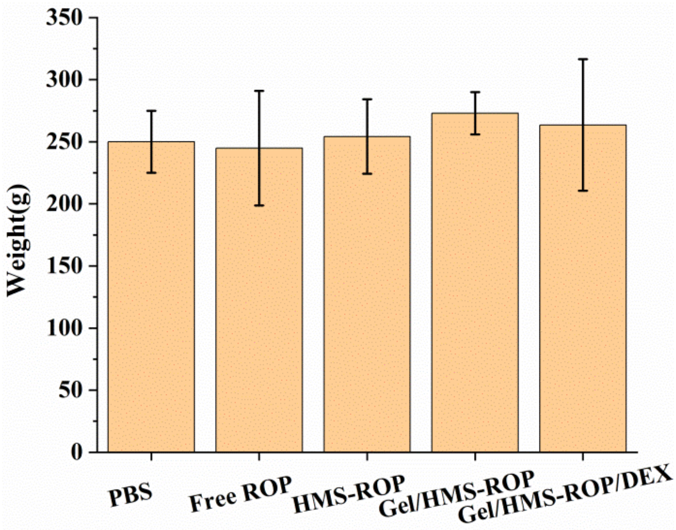

Figure S7 The body weight of SD rats before the experiment

Video.S1: Real-time preparation process of HMS-ROP in microfluidic chip

Video.S2: Testing the injectability of Gel/HMS-ROP/DEX composite based on mechanical testing machine

Video.S3: Hot plate experimental process of sensory block *in vivo*
